# Engaging Black youth in depression and suicide prevention treatment within urban schools: study protocol for a randomized controlled pilot

**DOI:** 10.1186/s13063-024-07947-8

**Published:** 2024-02-09

**Authors:** Michael A. Lindsey, Laura Mufson, Carolina Vélez-Grau, Tracy Grogan, Damali M. Wilson, Aaron O. Reliford, Meredith Gunlicks-Stoessel, James Jaccard

**Affiliations:** 1 Silver School of Social Work, New York University, 1 Washington Square North, New York, NY 10003 USA; 2 Department of Psychiatry, New York State Psychiatric Institute, Columbia University, 1051 Riverside Drive, New York, NY 10032 USA; 3 School of Social Work, Boston College, 140 Commonwealth Avenue, Chestnut Hill, MA 02467 USA; 4 McSilver Institute for Poverty Policy and Research, New York University, 708 Broadway, Fifth Floor, New York, NY 10003 USA; 5 Child & Adolescent Psychiatry, NYU Langone Health, 1 Park Avenue, 7th Floor, New York, NY 10016 USA; 6 Department of Psychiatry and Behavioral Sciences, University of Minnesota, 2025 East River Parkway, Minneapolis, MN 55414 USA

**Keywords:** Depression, Treatment engagement, Adolescents, Black youth, Intervention

## Abstract

**Background:**

Depression continues to be an ongoing threat to adolescent well-being with Black adolescents being particularly vulnerable to greater burdens of depression as well as lower mental health service utilization. Black adolescents are likely to have untreated depression due to social network influences, varied perceptions of services and providers, or self-stigma associated with experiencing depressive symptoms. Furthermore, if or when treatment is initiated, low engagement and early termination are common. To address this gap, a trial is being conducted to preliminarily test the effectiveness of an engagement intervention targeting Black adolescents with depression in school mental health services in New York City.

**Methods:**

A total of 60 Black middle and high school adolescents displaying depressive symptoms are equally randomized (based on school site) to the treatment arms. Both trial arms deliver *Interpersonal Psychotherapy for Depressed Adolescents* (IPT-A), a time-limited, evidence-based treatment for depression. Additionally, one arm pairs IPT-A with a brief, multi-level engagement intervention, the *Making Connections Intervention* (MCI), involving adolescents, caregivers, and clinicians. Outcomes of interest are group differences in depression and suicide ideation, adolescent and caregiver engagement, and mental health service use.

**Discussion:**

This trial will serve as an efficacy assessment of the MCI among a sample of Black adolescent students with depressive symptoms. Clinical and implementation results will be used to inform future research to further test the MCI intervention in a larger sample.

**Trial registration:**

Registered by ClinicalTrials.gov on May 3, 2019, identifier: NCT03940508.

**Supplementary Information:**

The online version contains supplementary material available at 10.1186/s13063-024-07947-8.

## Background

The burden of adolescent depression continues to grow. Across several federal data collection systems, about one in five adolescents, 20.9%, had experienced a major depressive episode [1]. This is a significant increase from previous reports in the decade prior that found 10% of adolescents suffer from depression [2]. Although prevalence of depression is similar among Blacks and Whites, the chronicity and burden of depression are greater for Blacks [3]. Black youth and adults report a higher prevalence of dysthymic disorder (now called persistent depression disorder in the DSM-5), the chronic nature of which may explain the increase in suicide among Black adolescents, from 6.7 per 100,000 in 2007 to 11.8 per 100,000 in 2017 [1, 4, 5]. A recent study also notes that suicide rates, a sequela of untreated depression, among Black youth have increased exponentially over time. From 2001 to 2017, rates increased among Black boys and girls, 60% and 182%, respectively [6]. The current study seeks to address these important, race/ethnicity-based disparities. The COVID-19 pandemic exacerbated existing systemic disparities in the United States [7–9]. Black youth were disproportionately impacted by the pandemic and experienced higher rates of depression during this time compared with White youth [7]. This study also aims to provide insight on the impact of the COVID-19 pandemic on Black youth mental health.

Depression among Black adolescents often goes untreated, and lack of treatment engagement is a factor [10]. Nationally, about half of adolescents with depressive disorders never receive mental health (MH) treatment for depression [11]. Data from the National Comorbidity Survey – Adolescent Supplement indicate that compared with their White counterparts, Black adolescents are significantly less likely to receive care for depression [11, 12]. Additionally, for many Black adolescents, premature treatment termination is the norm and poor engagement is a key influence. Engagement research, however, has failed to build a strong empirical base regarding the evidence-based strategies essential to facilitating treatment engagement for adolescents, in general, and Black adolescents, specifically [13]. Theoretically, engagement has both behavioral (e.g., attendance, in-session participation, homework completion) and attitudinal (e.g., emotional investment, commitment to treatment) dimensions [14]. Most evidence-based engagement programs in child MH services research target the caregivers of children with MH needs and only focus on the behavioral domain—attendance is the primary outcome [12–15]. Rarely has the attitudinal dimension been considered relative to clinical outcomes in MH treatment engagement, despite the empirical evidence that certain cognitive factors (e.g., attitudinal buy-in) explain 27–40% of variance in behavioral change [13, 16, 17]. This is true among caregivers of children with MH needs, and among adolescents, for which there is limited literature [18–20]. Black adolescents with depression and those displaying early signs of suicidal ideation, for example, experience stigma and negative perceptions of both mental illness, as well as treatment services that ultimately impact their service use [21–23]. The present study addresses mechanisms pertaining to the *under*-treatment of depression.

Black adolescents often seek professional help as a last resort for MH treatment, and social networks play a pivotal role in shaping their help-seeking behaviors [24]. When experiencing emotional or psychological problems, Black adolescents discuss their problems almost exclusively with their family and can receive messages consistent with not talking to “outsiders” about their MH problems [21]. Peers and friends also influence Black adolescents’ help-seeking behaviors, e.g., they fear friends would tease and make fun of them about MH treatment and are reluctant to tell them [14, 21]. MH help-seeking among Black adolescents, therefore, may be more stigmatizing and social networks are potentially not as supportive [10, 14, 21].

Black adolescents and their caregivers’ perceptual barriers, in particular MH stigma and distrust of therapists, further impede the use of formal service. Compared with Whites, a feeling of embarrassment about seeking treatment is more severe among Blacks, and Black adolescents often associate the use of MH treatment for depression with feeling “shame” [21, 24]. Black adolescents and their caregivers also tend to question therapists’ genuineness and suspect that therapists would not be able to solve their problems [21]. Their concerns based on the historical treatment of racial/ethnic and sexual minorities in treatment may be warranted [25]. Furthermore, there is a shortage of Black therapists, which has been shown to impact Black individuals’ willingness to pursue therapy [23]. This mistrust of therapists, thus, can prevent Black youth from seeking treatment [20, 23].

Enhancing treatment motivation may potentially combat adolescents’ resistance or ambivalence toward professional MH treatment. The MH services literature is clear: Motivation-based interventions that facilitate treatment engagement and/or reduce risky behaviors, including substance use, alcohol addiction, tobacco use, HIV, gambling, and eating disorders, have a proven efficacy [26–29]. Applications of motivation-based interventions to MH treatment have emerged more recently and show great promise for increasing service use among adolescents with depression and other mental disorders [30, 31]. Unfortunately, Black adolescents are underrepresented in these studies [32]. How best to enhance treatment motivation among Black adolescents may be more nuanced, particularly given that their influential social networks tend not to favor formal treatment use as a first option for care [20, 21, 23].

Caregivers’ perceived relevance of treatment also is a critical mediator in adolescents’ seeking of professional services [20]. For instance, in the face of their children’s MH problems, Black caregivers often perceive formal treatment as irrelevant and give primacy to the perspectives of their network members, especially given the shame accorded to depression and other mental illnesses [20, 21]. Consequently, Black families are more inclined to rely on informal support, such as family members and church, for addressing emotional or psychological struggles [14, 33–36]. To engage Black adolescents into MH treatment, their caregivers’ perceived irrelevance of treatment needs to be addressed relative to these more informal “treatment” options. Furthermore, research has also confirmed the positive effect of caregivers’ knowledge about MH services on children’s treatment attendance and in-session participation [37]. Critically, psychoeducation modifies caregivers’ expectations and prepares them for the treatment process [15]. In a systematic review assessing engagement practice elements for families in child MH services, psychoeducation was among the most frequently used engagement strategies (used in 42.6% of the studies), indicating that a significant portion of RCTs studying engagement feature this strategy for use with caregivers of youth. Thus, it is essential to include psychoeducation as part of an adolescent engagement intervention to improve treatment attendance and cognitive preparation, two prominent outcomes indicating engagement among caregivers [18].

Given the known limitations of evidence-based therapies for depression, particularly among Black youth, as well as the lower rates of treatment participation among those same youth, the current study (1) examines the effectiveness of the Making Connections Intervention (MCI) and (2) seeks to identify key mediators of both engagement and response to treatment for depression among Black youth. MCI builds on prior evidence-based strategies found to be efficacious in a comprehensive review of engagement interventions targeting families in children’s MH services [13, 18]. The review identified “common elements” of successful engagement interventions, however, mostly targeting adult caregivers of youth with mental health needs. The MCI is unique in its focus on adolescent treatment engagement. As a preliminary test of its effectiveness, the MCI is implemented as an additive intervention with Interpersonal psychotherapy for depressed adolescents (IPT-A), a model treatment protocol shown to be effective in treating adolescent depression [38–45]. The primary study aims are to (1) revise and enhance a brief engagement intervention for inner-city, Black youth treated for depression using IPT-A, building triadic relations between adolescents, parents/caregivers, and clinicians utilizing in-person sessions and personalized digital technologies, and (2) pilot test the effect of the MCI to improve engagement in IPT-A and, in turn, identify key mediators of both engagement and response to treatment for depression, as well as effects on suicide ideation.

## Methods

### Study design, setting, and participants

This pilot study features a 2-arm, cluster randomized control design, with randomization at the school level. Sixty eligible students with symptoms of depression are randomized between schools, in a 1:1 ratio, to receive the MCI + IPT-A enhanced intervention or IPT-A only. The study takes place at seven (7) public and charter middle and high school School-Based Mental Health Clinics (SBMHCs) located in Manhattan and Brooklyn in New York City, New York, USA. School sites were chosen based on racial/ethnic demographics provided by our partner agencies. The partnering schools have approximately similar percentages of male and female students. The students who attend these schools are generally from low-income families (i.e., live below the poverty line, receive Medicaid).

Prior to the COVID-19 pandemic, the study randomization was planned at the student level using a computer-generated allocation table. No planned restriction was utilized. Two participants were enrolled using this schema. With the uncharted transition to telehealth delivery in March 2020 and clinician apprehension of implementing new treatment options, in discussion with partner sites, the decision was made to randomize at the school level. This decision was also to prevent the possible contamination of MCI techniques being inadvertently used for IPT-A only participants. Clinicians at the assigned school sites were notified at the same time after their training was complete, ethics board approval was obtained, and the study launch had commenced. Efforts were not made to conceal the site assignment from participants, as they would know whether or not they took part in an MCI session as described in the consent documentation, and they would know whether they had an MCI session or began with session one of IPT-A.

**Fig. 1:**
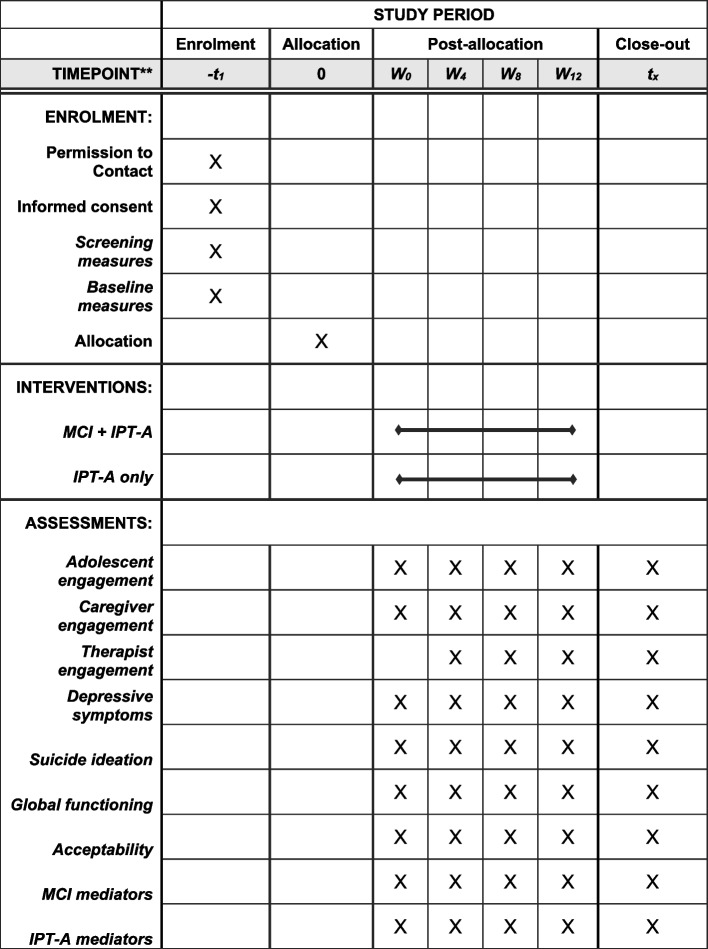
Example template of recommended content for the schedule of enrolment, interventions, and assessments. * *Recommended content can be displayed using various schematic formats. See SPIRIT 2013 Explanation and Elaboration for examples from protocols. **List specific timepoints in this row

Black adolescents with depressive symptoms in grades 6–12 (except 12th graders in their last semester) who are 12 years and older, English speaking, have received caregiver consent, and have assented to participate are included. Depressive symptoms are screened using a cut-off score of 10 or higher on the PHQ-9 and confirmed with a CES-DC score greater than 16 at the baseline assessment. Also, students who are on a stable dose of antidepressant medication, but still meet inclusion criteria, may be enrolled. Exclusion criteria are as follows: youth reporting active suicidal intent or plan; are intellectually disabled; have a life-threatening medical illness; a current primary substance abuse diagnosis in the moderate to severe range; schizophrenia, bipolar disorder, any evidence of psychosis; a primary diagnosis of anorexia; or are in active treatment for depression (excluding medication) at baseline. The school-based mental health clinicians deliver the MCI and IPT-A interventions, in-person at the youth’s school or virtually, via agency approved telehealth platforms. IPT-A replaces usual care for all enrolled participants. Participants may withdraw at any time and revert to usual care. Clinicians follow the safety and reporting procedures of their respective agencies if there is an increase in clinical symptoms Fig. 1.

### Recruitment and data collection

Most SBMHCs are staffed by one mental health clinician. To prevent contamination, treatment conditions are set at the school level, with each site designated as either an MCI+IPT-A or IPT-A only site. Thus, the study team and therapists are not blinded. The condition is also not blinded to participants as the treatment conditions are outlined in the consent/assent forms. Students are referred to the school clinic for possible treatment by teachers, staff, or self-referral, as normally occurs in most schools. The SBMHCs routinely complete PHQ-9 screenings for all behavioral health referrals. Students that come to school-based medical clinics initially receive the PHQ-2. Those that have scores indicating depression are then given the PHQ-9. Students that score 10 or higher are given a same day referral to the behavioral health clinic. A school mental health therapist receives the scores on the PHQ-9, and if there is an indication of adolescent depression (a cutoff score of 10), the therapist reaches out to the parent to discuss the youth’s entry into the treatment study. Adolescents who meet the cutoff score and the inclusion criteria are eligible for enrollment throughout the school year and summer. The school mental health therapist does the initial outreach to families for eligible students. Caregivers must consent to be contacted by the research staff via a Permission to Contact form sent by the clinician to the research assistant and filed securely on NYU Box. If the caregiver and youth agree to be contacted, a research staff member contacts the family regarding the study. Parental consent must be obtained prior to student assent. To respect caregivers’ and students’ time, research staff provide the option of obtaining consent via (1) telephone or (2) electronic survey. Paper copies are scanned on-site at NYU, with the physical copies stored in a locked cabinet and the digital copy stored on NYU Box. Participants are considered enrolled after completing the consent and assent process and the baseline survey. Research staff members conduct the consent and assessment procedures. No identifying images and personal or clinical details of participants are presented here or will be presented in reports of the trial results. Informed consent materials are attached as supplementary materials.

Student participants are assessed at baseline and after completion of the MCI session(s) and over the course of treatment at weeks 4, 8, and 12. Student screenings and assessments are conducted at the youth’s school or virtually via REDCap secure electronic surveys. Caregivers are assessed virtually on a parallel schedule: at baseline, post-MCI session (even if they are not in the MCI condition), and at weeks 4, 8, and 12 of the IPT-A treatment. Assessments are administered separately for student and caregiver to reduce bias. Participant responses are entered directly into REDCap by the research assistant administering the measures. All participants receive compensation in the form of gift cards for the time spent on assessments ($20 baseline and $15 subsequent assessment for caregivers; $15 baseline and $10 subsequent assessments for adolescents). There are no provisions for post-trial care or compensation. Therapists also complete the Clinical Global Improvement (CGI) scale, measures of adolescent and caregiver engagement, and treatment integrity measures associated with the MCI and IPT-A (Table 1).

**Table 1:** Constructs and measures

MCI mediators		
Construct	Measure	Data collection time point
Treatment barriers	Barriers for Adolescents Seeking Health (BASH) [46] (adolescent) (Cronbach’s *α*: 0.75 in the R21 study)	BASH: baseline, post-MCI sessions, weeks 4, 8, 12
Treatment attitudes	Child Help-Seeking Scale (HSS) [47] (adolescent) (Cronbach’s *α*: 0.70 in the R21 study); Attitudes Toward Psychological Help Scale (ATPHS) [48] (adolescent) (Cronbach’s *α*: 0.69 in the R21 study)	HSS: baseline, post-MCI sessions, weeks 4, 8, 12; ATPHS: baseline, post-MCI sessions, weeks 4, 8, 12
Treatment motivation	Stages of Change (SOC) [49–51] (adolescent) (Cronbach’s α: 0.75–0.87 in the R21 study)	SOC: baseline, post-MCI sessions, weeks 4, 8, 12
Alliance	Penn Helping Alliance (Haq-R) [52] (Adolescent) (Cronbach’s *α*: 0.89 in the R21 study)	Haq-R: post-MCI sessions, weeks 4, 8, 12
Treatment integrity	MCI Integrity Scale (therapist)	Completed after each session
Perceived relevance of treatment	Barriers to Treatment Participation Scale (BTPS) [53] (caregiver) (Cronbach’s *α*: 0.86)	BTPS: baseline, post-MCI sessions, weeks 4, 8, 12
Caregiver knowledge about depression	Understanding Mood Disorders Questionnaire (UMDQ) [54] (caregiver) (Cronbach’s *α*: 0.84–0.90)	UMDQ: baseline, post-MCI sessions, weeks 4, 8, 12
Caregiver knowledge about MH services	Therapy Survey (TS) [55] (caregiver) (Cronbach’s *α*: 0.52)	TS: baseline, post-MCI sessions, weeks 4, 8, 12
Social connectedness and social competence	Interpersonal Needs Questionnaire (INQ-15) [56] (adolescent) (Cronbach’s *α*: 0.75–0.90)	INQ-15: baseline, post-MCI sessions, weeks 4, 8, 12
**IPT-A mediators**		
Alexithymia	Toronto Alexithymia Scale--Externally-Oriented Thinking Subscale [57] (adolescent)	TAS-subscale: baseline, post-MCI sessions, weeks 4, 8, 12
Empathy	Empathy Questionnaire for Children and Adolescents [58] (adolescents)	EmQue-CA: baseline, post-MCI sessions, weeks 4, 8, 12
**Main study outcomes**		
Adolescent engagement	Progress of Treatment (POT) [59] (therapist)	POT: after each session (MCI+IPT-A)
Caregiver engagement	Engagement Measure (EM) [60] (caregiver, therapist) (Cronbach’s *α*: 0.98)	EM: weeks 4, 8, 12
Therapist engagement	Evidence-Based Practice Attitude Scale (EBPAS) [61] (therapist) (Cronbach’s *α*: 0.77)	EBPAS: pre-MCI session, post-MCI sessions, weeks 4, 8, 12
Depressive symptoms	Hamilton Rating Scale for Depression (HRSD) [62] (independent evaluator or research staff); Center for Epidemiologic Studies Depression Scale for Children (CES-DC) [63] (adolescent)	HRSD: screen, baseline, post-MCI session, weeks 4, 8, 12; CES-DC: screen, baseline, weeks 4, 8, 12
Suicide ideation	Suicide Ideation Questionnaire-Junior (SIQ-JR) [64]; Columbia-Suicide Severity Rating Scale (C-SSRS) [65]	SIQ-JR: baseline, weeks 4, 8, 12; C-SSRS: baseline, weeks 4, 8, 12
Global functioning	Global Functioning Scale for Children (C-GAS) [66] (independent evaluator or research staff); Clinical Global Improvement (CGI) [67] (independent evaluator or research staff)	C-GAS: baseline, weeks 4, 8, 12; CGI: baseline, weeks 4, 8, 12
**Covariates**		
Demographics	Demographic Form (DF) (caregiver)	DF: baseline

During data collection, a participant identification number is assigned to each participant and indicated on research forms so that it is the only identifying link to assessment data. It is this identification number that appears on all study materials, with the exception of consent and assent forms, both of which are stored in a separate location from treatment and assessment files. Access to consent forms, assent forms, demographic information, audio tapes of sessions, and caregiver assessment materials is limited to the PI and research staff at NYU. In addition to research staff, school mental health therapists have access to student assessments. All data from this research study are stored on the university’s secure servers as password protected files.

Research team members and school-based therapists collaborate on outreach and retention efforts. For example, both outreach to participants using telephone calls, in addition the research team uses app notifications, while the therapists provide in-person reminders (clinicians) for therapy sessions and assessment appointments.

This study does not have a data safety monitoring board. The study is subject to random audits by the IRB; one was conducted in 2021. No biological specimens are collected during this research.

### Intervention

IPT-A is an intervention teaching communication and problem-solving strategies, specifically, targeting interpersonal problems characteristic of adolescent depression (grief, role disputes, role transitions, interpersonal deficits). It is divided into the following three phases:

1. Initial phase—confirmation of depression diagnosis, psychoeducation, review of significant interpersonal relationships, identification of problem areas;
2. Middle phase—identification and practice of strategies that help adolescents resolve their interpersonal difficulties as well as regulate their emotions through learning to express them more easily to avoid build up and feelings of being overwhelmed; and
3. Termination phase—review of new interpersonal skills, fostering generalization to future situations, clarification of need for further treatment).

IPT-A has been demonstrated to be efficacious and effective in alleviating depression symptoms and improving functioning [68]. Twelve sessions are completed over 12 weeks in a weekly 30–45-min individual therapy format. Though sessions take place with the student, there is the possibility for concurrent sessions with the caregiver, as needed.

The MCI is a theory-driven, 1–2 session intervention designed to improve engagement, perceived relevance, and treatment satisfaction among depressed, Black adolescents. It builds on prior evidence-based strategies of engagement in children’s MH services. The MCI reduces negative perceptions of help seeking. A focus of the intervention is reducing perceived barriers (such as mistrust, help-seeking efficacy, shame) to MH treatment for Black adolescents and their caregivers. The MCI helps reconcile the positive consequences and disadvantages of treatment, thereby enhancing treatment motivation.

Both students and caregivers assigned to the MCI + IPT-A group attend one or two MCI sessions lasting approximately 45 min prior to initiating IPT-A. Clinicians also meet with students for a session focused on the mobile app and a review of the app’s features. Of note, if there is no progress on the Stages of Change measure from baseline assessment to post-MCI assessment; then, a second MCI session is determined by the research team. Adolescent participants then receive IPT-A for 12 sessions. Thus, MCI coupled with IPT-A consists of a total of 13 to 14 sessions.

### Ethical considerations

As this study focuses on depressed youth, suicide ideation is not unexpected. Research staff are trained in how to handle participant disclosure of suicidal ideation during assessments. If a student has active ideation, the study PI and therapist are notified, and the therapist follows their respective agency procedures on response and safety. The IRB is notified of any adverse events. Additionally, any changes to the study protocol are submitted and reviewed by the IRB before being implemented. Any deviations from the approved protocol are reported to the IRB using their internal reporting system.

There are no officially titled committees associated with this study. The PI and research team meet twice monthly to discuss recruitment, implementation, and study progress. Additionally, the investigators and research team meet monthly with direct service provider partner agencies. The research assistant, project director, and clinical supervisors meet weekly with the school-based therapists by treatment arm: one group supervision for MCI+IPT-A, one group supervision for IPT-A only. There is no Stakeholder and Public Involvement Group for this study.

### Analysis plan

Two types of analyses are planned. The first will be for treatment-based outcome differences between the IPT-A+MCI and the IPT-A only conditions. These will take the form of single degree of freedom contrasts between the two groups comprising the pilot randomized trial to demonstrate intervention effects. Of primary interest will be group differences on depression and suicide ideation, our primary outcomes, using the baseline measure (if available) as a covariate. With a planned *N* of 30 per group, for two tailed comparisons at alpha = 0.05 and power of 0.80, we anticipate an effect size sensitivity of *d* = 0.75 and, after covariate control of the baseline outcome measure and covariates to adjust for possible imbalance, effect size sensitivity of *d* = 0.55 (see McClelland, 2000, and Raush, Maxwell, and Kelley, 2003, for a discussion of using covariates to increase statistical power and effect size sensitivity) [69, 70]. The second set of planned analyses are bivariate and small multivariate regressions to identify mediators that would have promise for pursuit in a larger trial in the future. It also will sensitize us to components of the MCI that may need revision. For an *N* of 60, the effect size sensitivity for a bivariate regression for a two tailed test with power = 0.80 is approximately *r* = 0.35. No subgroup or interim analyses are planned given the sample size. Missing data will be addressed using multiple imputation with variants of chained equations [71, 72]. With respect to treatment dropouts and non-adherence, we will perform both intent to treat and per protocol analyses, with preference being for the latter given the early stages of the research. Condition imbalance for the per protocol sample, should it occur, will be addressed through direct covariate control or IPTW strategies [73].

## Discussion

A major implementation change was needed to address the shift to remote schooling and the closure of school health clinics due to SARS-CoV-2 (COVID-19) in March 2020. This study was originally conceptualized with consent, assent, and surveys procedures taking place in the SBMHC where the participants also receive MH services. The research team worked with our agency partners to update our procedures while they balanced the shift to telehealth delivery for services. Telephone consent and assent and telephone or virtual surveys were designed and remain in effect to allow for more flexibility for students and caregivers. However, there are notable disadvantages to remote procedures. First, it is unclear to what extent the switch to telehealth impacts engagement, as only two participants were enrolled before the shutdown. Those participants were subsequently discontinued and transitioned to usual care. Their therapists did not feel confident managing both their first study cases and the switch to a new telehealth platform and workflow at the same time. Second, “Zoom fatigue” secondary to online schooling may have impacted participants as many began to turn their cameras off during assessments or requested telephone calls instead [74, 75], which has continued with the return to in-person schooling. This is consistent with recent work demonstrating the relationship between camera usage and Zoom fatigue [75]. Finally, in the transition to remote participation, some students reported constraints on privacy for sessions and surveys at home as compared with being in a school setting. While privacy issues were discussed during consent and assent processes, privacy challenges remain.

The ultimate goal of this study is to enhance engagement in mental health treatment among Black youth, through examination of the effectiveness of a brief engagement intervention with target youth experiencing depressive symptoms. As real-world challenges, such as the COVID-19 pandemic emerged, additional design and implementation adaptations were needed. With that, the study may also provide additional insight on the impact of remote versus in- person delivery on engagement in psychotherapy in the school-based setting.

## Trial status

Protocol version: 12/06/2022

Recruitment start date: 1/02/2020

Anticipated recruitment end date: 10/01/2023

### Supplementary Information

**Additional file 1.**

**Additional file 2.**

## Data Availability

All data collected in this study will be made available to the scientific community for educational, research, and non-profit purposes. Findings will be disseminated to community partners, at the local level through a written summary or presentations of results. Public engagement events, conference posters, and/or oral presentations will take place at a national level. After the final data analyses are complete, findings will be published.

## Abbreviations

EBT: Evidence-based therapy
IPT-A: Interpersonal psychotherapy for depressed adolescents
LGBTQ: Lesbian, gay, bisexual, transgender, queer/questioning
MCI: Making Connections Intervention
MH: Mental health
SBMHC: School-Based Mental Health Clinics
US: United States
