# Correction: Engaging Black youth in depression and suicide prevention treatment within urban schools: study protocol for a randomized controlled pilot

**DOI:** 10.1186/s13063-024-08084-y

**Published:** 2024-04-11

**Authors:** Michael A. Lindsey, Laura Mufson, Carolina Vélez‑Grau, Tracy Grogan, Damali M. Wilson, Aaron O. Reliford, Meredith Gunlicks‑Stoessel, James Jaccard

**Affiliations:** 1 Silver School of Social Work, New York University, 1 Washington Square North, New York, NY 10003 USA; 2 Department of Psychiatry, New York State Psychiatric Institute, Columbia University, 1051 Riverside Drive, New York, NY 10032 USA; 3 School of Social Work, Boston College, 140 Commonwealth Avenue, Chestnut Hill, MA 02467 USA; 4 McSilver Institute for Poverty Policy and Research, New York University, 708 Broadway, Fifth Floor, New York, NY 10003 USA; 5 Child & Adolescent Psychiatry, NYU Langone Health, 1 Park Avenue, 7Th Floor, New York, NY 10016 USA; 6 Department of Psychiatry and Behavioral Sciences, University of Minnesota, 2025 East River Parkway, Minneapolis, MN 55414 USA

**Correction** **:** **Trials 25, 112 (2024)**

**https://doi.org/10.1186/s13063-024-07947-8**

Following publication of the original article [1], we have been notified that the generic SPIRIT diagram was presented as Figure 1, instead of the study’s specific figure.

Originally published Figure 1:


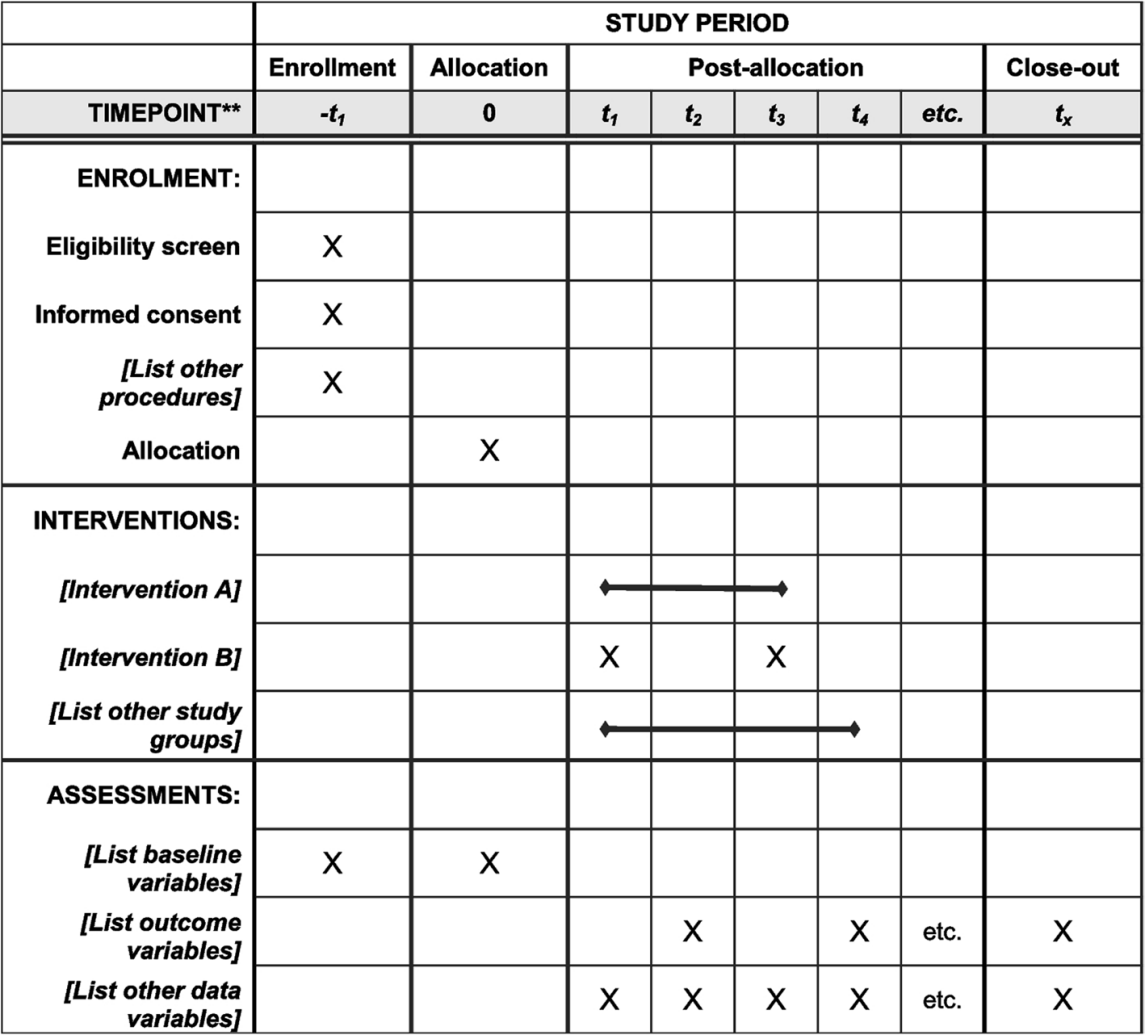


Correct Figure 1:


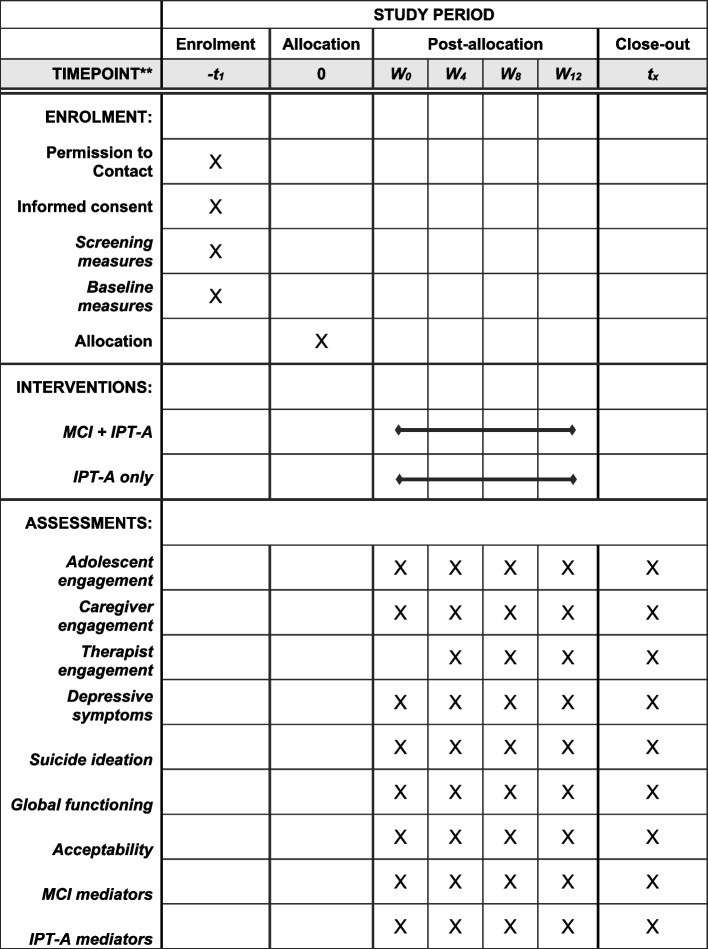


The original article has been corrected.
