# Supplementary material for: Engaging Black youth in depression and suicide prevention treatment within urban schools: study protocol for a randomized controlled pilot
Source: Trials. 2024 Feb 9;25:112. doi: 10.1186/s13063-024-07947-8 (PMC10854091; doi:10.1186/s13063-024-07947-8)
Supplement: Supplementary file 1 — Additional file 1. [file 13063_2024_7947_MOESM1_ESM.zip › Brany Approved/BRANY Approved- ICF-HSAssent (4)R2.pdf]

SILVER SCHOOL OF SOCIAL WORK
1 Washington Square North
New York, NY 10003 USA

Research Assent/Consent Form – High School Students

We want to tell you about a research study we are doing. A research study is a way to learn information about something. We would like to find out more about how students and their families feel when they first begin talking to a counselor. You have been asked to be in this study because you have been referred to work with a counselor at school.

Purpose of Study

This study will look into how to help Black students with depression get the most out of treatment. The study team will investigate how to find the best way to deliver evidence-based treatment. All of the students and parents who participate in this study will fill out some questionnaires ("surveys" for the middle school version). All students will receive a 12 session treatment for depression called IPT-A. Depending on which school you attend, half of the students will begin their treatment with one family session called the Making Connections Intervention or MCI. The MCI is an extra session to help you and your parent(s) understand parts of therapy you may not know about and to answer any questions or concerns you may have about starting therapy. You will also be asked to participate in an interview. If you agree to join the study, we will first ask you and your parent(s) a few questions to help the counselor decide what we will talk about during the MCI session. If you do not receive the MCI session, you will begin your first session with the IPT-A.

There will be about 20 parent-student pairs in each year of the study, totaling 60 pairs over the 3 years.

Study Procedures

This study is an added part to go with regular counseling given by the school mental health counselor at your school. For this study, one of our research team members will ask you and your parent(s) a series of questions from a few questionnaires to help the counselor understand you and your parent's feelings and thoughts about beginning therapy. These questions will be asked in person during one of your first meetings with the counselor. The questions will be given to you and your parent separately and will take about 45 minutes. One of our research team members will meet with you to ask more questions after your first session (the MCI session) and every 4 weeks until you finish the study (weeks 4, 8, and 12) but these meetings will be shorter and only take 20-25 minutes.

After completing these questions, the counselor will schedule the MCI session with you and your parent(s). This session will take about one hour and will happen during the school day. There are some parts of the MCI session where your parent(s) may choose to leave the session to allow you to talk with the counselor privately, that is up to your parent(s) to choose what you and your parent(s) are most comfortable with. After the MCI session, therapy will begin just like it normally would with the counselor.

There may be times where a session will need to be done remotely. The clinician or research team member will work with you to schedule the best time for a telehealth or remote assessment session.

You will also be asked to complete one interview at or after Week 4 of the study.

As part of the study, all of the sessions will be tape recorded (audiotaped) unless your caregiver chose to opt out. The sessions are audiotaped because the research team wants to make sure you are given the best services and because they want to know what topics are talked about the most in your sessions. These tapes will only be listened to by the researchers and by the supervisors of the counselors in the

BRANY
IRB APPROVED
02/13/2023

school clinic. The tapes will be kept in a locked cabinet for up to 5 years at the New York University, School of Social Work, McSilver Institute for Poverty Policy and Research; however, if you and/or your caregiver at any time decide you no longer want your sessions recorded and saved, the recordings will be destroyed. You also have the right to review all or any portion of the tape.

Finally, we would like to ask for your permission to obtain the results from the initial screening tool (PHQ-9) that your school mental health counselor used to determine whether you have symptoms consistent with depression. This will help the research team to understand your feelings prior to your enrollment in the study. By signing this consent, you will give us permission to access the results from this screening tool.

Risks

Being in the study will probably not cause any problems for you or your family. The meetings with the clinicians and research team will take some time. It is possible that they could make you feel upset or tired. If this happens, you can choose not to answer specific questions or ask to have the interview stopped at any time. In talking about particular problems, you may not find this treatment helpful or you may begin to feel worse. If so, you will be evaluated and referred for a different type of treatment either in the school-based mental health program or another agency. It is possible that others may find out private or confidential information about you or your family through participating in this project but we will make sure all sessions take place behind closed doors where no one can hear what you or your family says and all of the surveys you fill out will be kept in a locked file cabinet in a locked office at New York University. If you take part in a telehealth session, you may have additional privacy concerns while at home. You should feel free to discuss any concerns with your clinician or the research team member. They will work with you to find a solution, including rescheduling.

New York law requires us to report any cases of suspected child abuse or neglect to state officials. Also, most of the things you will talk about with the therapist will not be told to your parent(s)/guardian(s)). But if the therapist thinks you are in danger of hurting yourself or someone else, they will have to tell your parent(s)/guardian(s). If the therapist has to tell your parent(s)/guardian(s) about something you have talked about, they will talk to you about it before they talk to your parent(s)/guardian(s).

There is a chance you may not feel better from therapy but may feel worse. If this happens the counselor at your school will help you and your parents to get the kind of help that you need.

Benefits

This study may be good for you if it makes working with the therapist easier. Also, the study may help other teenagers in the future, by learning more about how to help teenagers who are just starting therapy.

Alternative Treatments or Alternative to Study Participation

You do not have to be in this study in order to get help from the counselor at your school. If you do not want to be in the study, you can still go to the school mental health counselor where you will be treated like any other student who goes there for help.

You do not have to join this study. It is up to you. You can say okay now, and you can change your mind later. All you have to do is tell us. No one will be mad at you if you change your mind.

Anything we learn about you from this study will be kept as secret as possible.

BRANY
IRB APPROVED
02/13/2023

SILVER SCHOOL OF SOCIAL WORK
1 Washington Square North
New York, NY 10003 USA

BRANY
IRB APPROVED
02/13/2023

Questions

If there is anything about the study or your participation that is unclear or that you do not understand, if you have questions or wish to report a research-related problem, you may contact Dr. Michael Lindsey at 212-998-5927, Michael.Lindsey@nyu.edu, or 708 Broadway, 5th Floor, New York, NY 10003.

If you have any questions about your rights as a research subject or complaints regarding this research study, or you are unable to reach the research staff, you may contact a person independent of the research team at the Biomedical Research Alliance of New York Institutional Review Board at 516-318-6877. Questions, concerns or complaints about research can also be registered with the Biomedical Research Alliance of New York Institutional Review Board at www.branyirb.com/concerns-about-research.

Before you say yes to be in this study, we will answer any questions you have.

If you want to be in this study, please sign your name. You will get a copy of this form to keep for yourself.

____
(Print your name here)

____________________________________ ________________
(Sign your name here to assent if you are **17 years old or younger**) Date

____________________________________ ________________
(Sign your name here to consent if you are **18 years old or older**) Date

____
Printed name of person obtaining assent/consent

____________________________________ ________________
Signature of person obtaining assent/consent Date

BRANY
IRB APPROVED
02/13/2023
