# Supplementary material for: Engaging Black youth in depression and suicide prevention treatment within urban schools: study protocol for a randomized controlled pilot
Source: Trials. 2024 Feb 9;25:112. doi: 10.1186/s13063-024-07947-8 (PMC10854091; doi:10.1186/s13063-024-07947-8)
Supplement: Supplementary file 1 — Additional file 1. [file 13063_2024_7947_MOESM1_ESM.zip › Brany Approved/BRANY Approved- ICF-Parent-NIMH-MCI IPT-A (3)R2.pdf]

SILVER SCHOOL OF SOCIAL WORK
1 Washington Square North
New York, NY 10003 USA

Parental Consent

This is a research study, participation is voluntary. You may ask questions at any time.

Key Information Statement:

The purpose of the study is to learn about ways people who work in school clinics can help Black adolescents who are depressed or sad. The study will examine how best to connect Black adolescents with depression to treatment in clinically meaningful ways, and how best to deliver evidence-based treatment to them through school-based services. Participants will be asked to take part in counseling treatment sessions with the school mental health counselor once a week during school, complete questionnaires, and participate in an interview at Week4 or later in the study. Study participation will last about 12 weeks. Participation in the interview will be one approximately 45-60-minute session. Study participation will last about 12 weeks. Participation in this research is completely voluntary. There is a risk that your child's problems may not get better or your child could feel worse, in which case we will refer your child for a different type of treatment. Although loss of confidentiality is a potential risk of taking place in this study, there are procedures in place to keep the counseling sessions private and the study records confidential and secure.

If you are interested in learning more about this study, please continue to read below.

Purpose of the Study

You and your child have been invited to take part in a research study to learn more about ways people who work in school clinics can help Black adolescents who are depressed or sad. This study is funded by the National Institute of Mental Health and will be conducted by Dr. Michael A. Lindsey of the McSilver Institute for Poverty Policy and Research, NYU Silver School of Social Work.

This study is being conducted with the School Health Program—Family Health Centers at NYU Langone at several New York City schools and with the Institute for Family Health (IFH) at two Harlem Children's Zone (HCZ) schools. NYU Langone's and IFH's clinicians will deliver the mental health services for the study because they are the mental and behavioral health providers for your child's school. There will be about 20 parent-student pairs in each year of the study, totaling 60 pairs over the 3 years. All of the students who participate in this study will be given a counseling treatment for depression called the Interpersonal Psychotherapy for Depressed Adolescents (IPT-A), while some parent-student pairs may receive the Making Connections Intervention (MCI). The MCI is an extra session(s) to help you and your child understand parts of therapy you may not know about. You have been asked to be in this study because one of the school staff members has reported some behaviors and feelings that show that your child may be

BRANY
IRB APPROVED
02/13/2023

sad or depressed. Or, your child indicated on an assessment that he or she may have depression symptoms.

We will first ask you and your child a few questions to determine if s/he qualifies to participate in this study. If s/he does not, s/he can always see the school counselor and we will give you and your child information on other mental health resources and agencies you and your child may contact for care.

Study Procedures

This study provides counseling for depression with the school mental health counselor. There are different types of help for depression and sometimes it just gets better on its own. If your child qualifies and you and your child agree to be in the study, you will be randomly assigned (like by the flip of a coin) based on the school your child attends to participate in one of two groups:

- Group in which you and your child attends 1-2 MCI sessions, and then your child receives 12 IPT-A sessions
- Group in which your child begins 12 IPT-A sessions

Depending on the group you are in, you will be asked to do the following:

1. You and your child may be asked to attend the one or two MCI session(s) which take approximately 45-60 minutes. The MCI session focuses on how to help parents/legal guardians and adolescents understand more about the therapy process prior to beginning counseling. The clinician will also review an app your child can use throughout treatment during this session. You and your child may also receive an invitation to interview to discuss your experiences with treatment so far.
2. Or, your child may begin to receive the IPT-A (or begin after the MCI) for 12 more sessions that last about 45 minutes each. The MCI and IPT-A meetings will take place during school once a week; if sessions are missed due to absence or school not being in session these sessions will be made up in subsequent weeks.
3. You and your child will each be given a series of questionnaires throughout the study by one of our research team members. The purpose of these questionnaires is to see how you and your child are doing, and how your child is feeling, how s/he is doing in school, how s/he is getting along with people, and any illnesses in your family. Your child will be given these questionnaires at the beginning of the study, and possibly after the MCI session(s), and at weeks 4, 8, and 12. You (parent/legal guardian) will be given these questionnaires at the beginning and end of the study.
4. You and your child will participate in an interview.
5. If your child attends treatment and continues to feel sad when it is over, his/her counselor will talk with you and your child about continuing therapy beyond the research study.

BRANY
IRB APPROVED
02/13/2023

SILVER SCHOOL OF SOCIAL WORK
1 Washington Square North
New York, NY 10003 USA

All parts of the study, including therapy sessions and assessments, can be delivered by telehealth if needed. Depending the situation at your child's school and/or government recommendations or requirements, part or all of the study may proceed via telehealth. The clinician and research team will work with you and your child to ensure the safest delivery method.

1. Students at NYC DOE schools served by NYU Langone clinicians will receive telehealth services via the WebEx platform. Students at HCZ schools served by IFH will receive telehealth services via the Doxy.me platform. Clinicians will follow their respective agencies guidelines regarding these platforms.

    a. Audio of telehealth sessions will be captured as with in-person sessions. No video or video-and-audio of sessions will be saved or used for the study.

2. Independent evaluators will use Zoom to conduct remote assessments.

3. Participants can opt for telephone administration of sessions or assessments due to technology access, technology services interruption or errors, or for privacy. Telephone sessions may not be able to be recorded.

Clinically Relevant Research Results

Study results will be published on ClinicalTrials.gov and shared with the National Institute of Mental Health's Data Archive as required by the NIH. We also plan to publish study results in peer-reviewed journals. Participants may request information about study results, including their individual results, from the principal investigator, Dr. Michael Lindsey, after the study is completed.

Audiotaping

As part of the study, all therapy sessions will be audio-recorded (voice). The sessions are recorded because the research team wants to be able to know what kinds of help are being given to teenagers in the study. These recordings will only be listened to by the researchers and by the supervisors of the counselors in the school clinic; no more than 2 to 3 people will listen to the recordings. Interviews will be audio recorded; no video will be recorded or retained. The recordings will be kept on a secure computer network that can only be accessed by password for up to 5 years; however, if you and/or your child at any time decide you no longer want your sessions recorded and saved, the recordings will be destroyed. You may opt out of audio recordings when you sign the form. You also have the right to review all or any portion of the tape.

Follow-up Contact

If you decide to be in the study, you also give the research staff permission to get information about your child from any agency or mental health professional to which h/she is referred for treatment during the time of the study.

BRANY
IRB APPROVED
02/13/2023

How much time will participation involve?

If your child meets study inclusion criteria and you agree to take part in this research, your child will participate in the screening session, MCI sessions(s), and the post-MCI assessment. You may also be asked to participate in the IPT-A sessions. . You will work with the research team member to schedule convenient times to meet. You should also feel free to ask any questions or address any concerns that you might have now or at any point during the study.

The first meeting to complete the study questionnaires will take approximately 45 minutes and will be conducted with you and your child together although the questionnaires will be given in separate rooms. The last meeting with your child will also take approximately 45 minutes. All meetings to complete the questionnaires with your child during the middle of the study will take approximately 20-25 minutes. You/your child will be compensated each time questionnaires are completed (at the beginning, during participation in the MCI and IPT-A, and at the end of participation). Finally, we would like to ask for your permission to obtain the results from the initial screening tool (PHQ-9) that the school mental health counselor used to determine whether your teen has symptoms consistent with depression. This will help the research team to understand the teen's feelings prior to the study. By signing this consent, you will give us permission to access the results from this screening tool.

Potential Risks/Discomforts

Being in the study is not expected to cause any problems for you or your family. The meetings with the clinicians and research team will take some time. It is possible that they could make you or your child feel upset or tired. If this happens, you and your child can choose not to answer specific questions.

In talking about particular problems, it is possible that your child's problems may not get better or your child could feel worse. If so, your child will be evaluated and referred for a different type of treatment. Although loss of confidentiality is a potential risk of taking place in this study, all in-person sessions/meetings will take place behind closed doors at the school where your child attends. There may be additional privacy concerns for telehealth sessions. Participants can discuss their concerns with the clinician or independent evaluator before beginning a session to ensure that the participant is comfortable continuing and to discuss possible solutions (for example, moving a session to telephone while the participant goes for a walk, etc.).

Further, all materials gathered will be kept in a locked file cabinet in a locked office accessible only to research staff.

Potential Benefits

This study may be good for your child if his/her depression gets better. Also, the study may help other teenagers in the future by learning more about how to help teenagers who are depressed

BRANY
IRB APPROVED
02/13/2023

and how to give this help in school clinics. You need to decide if your child's participation in this research study is in your child's best interest.

Payment to Participants

You will be given gift certificates for your time when you either go in person or answer on the phone the questions from the research staff. The gift certificates are to recognize your participation in filling out the questionnaires, not for the treatment itself. For the initial screening/baseline session Parents or legal guardians will receive a $20 gift certificate and students will receive a $15 gift certificate. For completion of the post MCI-session assessment, students and Parents or legal guardians (if Parents or legal guardians are involved) will each receive $10 gift certificates. At the completion of weeks 4, 8 and 12 assessments, students will receive $10 gift certificates (after each assessment). This is a total of $135 for each family ($80.00 total paid to the parent/legal guardian and $55.00 paid to the child, respectively, if all sessions are completed).

Confidentiality, Access to Records, and Authorization to Use and Disclose Personal Health Information

All forms that you or your child complete (records, audio recordings and any other research material) will be stored in locked filing cabinets or secure servers so that it is kept private. This information will not be shared with school officials. These researchers will only disclose your personal information, including research study records and personal health information, to the research staff, and to official Federal, State and Institutional regulatory personnel. These personnel may review records as part of the routine reviews of research projects.

Efforts will be made to limit your personal information including research study records to people who have a need to review this information. We cannot promise complete secrecy. Persons and organizations that may inspect and copy your information include the Institutional Review Board (IRBs) of New York University, New York State Psychiatric Institute (NYSPI), the Biomedical Research Alliance of New York, NYU Langone, the New York City Public Schools, the Institute for Family Health, Harlem Children's Zone, and the National Institute of Mental Health, who sponsored and provided the funding for this study.

The monitors, auditors, the IRBs (at New York University, NYSPI, the Biomedical Research Alliance of New York, NYU Langone, the New York City Public Schools, the Institute for Family Health), and the National Institute of Mental Health will be granted direct access to your study records and medical records for verification of the research procedures and date. These groups may then also share your personal health information, in which case it may no longer be covered by federal privacy laws. However, these groups are committed to keeping your personal health information confidential.

Additionally, we will remove identifiers from the identifiable private information you or your child provide for this research and provide this de-identified study information to the National Institute of Mental Health for use in future research studies without obtaining additional consent from you or your child.

BRANY
IRB APPROVED
02/13/2023

Study records will be considered confidential, and the participant's name will not be used in reports or publications. The data from the study may be published. However, you will not be identified by name. People designated from the institutions where the study is being conducted and people from the sponsor will be allowed to inspect sections of your medical and research records related to the study. Everyone using study information will work to keep your personal information confidential. Your personal information will not be given out unless required by law. You have the right to review and copy your health information, but you may not be allowed to do so until after the research is completed. By signing this document you are giving permission to use and share your research study records, including your personal health information, for the purposes described above.

This authorization does not have an expiration date. You have the right to cancel your consent at any time by giving written notice to the researchers. If you withdraw your permission, you will not be able to continue in this study, but you will not lose access to treatment or other benefits to which you are entitled. When you withdraw your permission, no new health information about you will be gathered after that date. Information that has already been collected may still be used and given to others.

This research is also covered by a Certificate of Confidentiality from the National Institutes of Health. Researchers with this Certificate will not disclose or use information that may identify you in any federal, state, or local civil, criminal, administrative, legislative, or other action, suit, or proceeding, even if there is a court subpoena.

Exceptions include:

- A federal, state, or local law requires disclosure.
- Your explicit approval for the researchers to release your name and/or personally identifiable information.

Limits to privacy/confidentiality: New York law requires us to report any cases of suspected child abuse or neglect to state officials. Also, most of the things your child will talk about with the therapist will not be told to you (parent(s)/guardian(s)). But if the therapist thinks that the child is in danger of hurting her/himself or someone else, they will have to tell you. If the therapist has to tell you about something your child has talked about, they will talk to the child about it before they talk to you.

A description of this clinical trial will be available on *http://www.ClinicalTrials.gov*, as required by U.S. Law. This Web site will not include information that can identify you. At most, the Web site will include a summary of the results. You can search this Web site at any time.

Alternative Treatments or Alternative to Study Participation

The study procedures differ from standard treatment in the following way: An existing treatment, the IPT-A, will be used in a new manner. In addition to the standard IPT-A treatment, participants in this study also receive the MCI as an extra one or two sessions.

BRANY
IRB APPROVED
02/13/2023

Your child does not have to be in this study in order to get help for being depressed or sad. If you or your child does not want to be in the study, s/he can still go to the school mental health counselor where s/he will be treated like any other student who goes there for help. If later on s/he seems to feel worse than s/he does now, you or your child can talk to a doctor or mental health person to get help or information about treatment. This may include taking medicine if prescribed by the doctor or therapist.

Right to Withdraw

Participation in this research study is voluntary. You do not have to take part in this research. If you do not participate or decide to stop participating, there will be no penalty to you. If you or your child decide not to participate, or if you or your child later decide to stop participating, you and your child will not lose any benefits to which you or your child are otherwise entitled. A decision not to participate will not affect treatment with the school mental health counselor. If you or your child need to stop taking part, if you have questions, concerns, or complaints, or if you need to report a medical injury related to the research, please contact the investigator, Dr. Michael Lindsey at the Silver School of Social Work, 212-998-5927.

If you withdraw from this study, already collected data may not be removed from the study database. You will be asked whether the investigator can collect data from your routine medical care provider. If so, then your records may be reviewed. If you do not give permission to access your records, then the research team will not access them.

Further Questions

If there is anything about the study or your participation that is unclear or that you do not understand, if you have questions or wish to report a research-related problem, you may contact Dr. Michael Lindsey at 212-998-5927, Michael.Lindsey@nyu.edu, or 708 Broadway, 5th Floor, New York, NY 10003.

If you have any questions about your rights as a research subject or complaints regarding this research study, or you are unable to reach the research staff, you may contact a person independent of the research team at the Biomedical Research Alliance of New York Institutional Review Board at 516-318-6877. Questions, concerns or complaints about research can also be registered with the Biomedical Research Alliance of New York Institutional Review Board at www.branyirb.com/concerns-about-research.

You have received a copy of this consent document to keep.

BRANY
IRB APPROVED
02/13/2023

SILVER SCHOOL OF SOCIAL WORK
1 Washington Square North
New York, NY 10003 USA

Agreement to Participate

and

Authorization to Use and Disclose Personal Health Information

____
Subject's Printed Name

____
Printed Name of Parent or Legal Guardian

____________________________________ ______________________
Signature of Parent or Legal Guardian Date

□ I do not want my child's sessions audio recorded.

____
Printed Name of Person Obtaining Consent

____________________________________ ______________________
Signature of Person Obtaining Consent Date

- Informed consent was obtained by telephone.
- A copy of the consent form has been emailed to the caregiver.
- A copy of the consent form has been mailed to the caregiver.

BRANY
IRB APPROVED
02/13/2023

SILVER SCHOOL OF SOCIAL WORK
1 Washington Square North
New York, NY 10003 USA

MCI Headway App Privacy Policy

Your privacy is important to us. It is Ker-twang's policy to respect your privacy regarding any information we may collect from you across our mobile application ("app") and other sites we own and operate.

We only ask for personal information when we truly need it to provide a service to you. We collect it by fair and lawful means, with your knowledge and consent. We also let you know why we are collecting it and how it will be used.

We only retain collected information for as long as necessary to provide you with your requested service. What data we store, we will protect within commercially acceptable means to prevent loss and theft, as well as unauthorized access, disclosure, copying, use or modification.

We do not share any personally identifying information publicly or with third-parties, except when required to by law.

Our app may link to external sites that are not operated by us. Please be aware that we have no control over the content and practices of these sites, and cannot accept responsibility or liability for their respective privacy policies.

You are free to refuse our request for your personal information, with the understanding that we may be unable to provide you with some of your desired services.

Your continued use of our app will be regarded as acceptance of our practices around privacy and personal information. If you have any questions about how we handle user data and personal information, feel free to contact us.

This policy is effective as of 16 June 2020.

BRANY
IRB APPROVED
02/13/2023
